# Editorial

**Published:** 1839-12-07

**Authors:** 


THE MEDICAL EXAMINER.

PHILADELPHIA, DECEMBER 7, 1839.

### EPIDEMIC DISEASES OF THE U. STATES.

WE are much gratified to find that the desire which we expressed a short time since, to receive details of the different epidemic diseases which have prevailed throughout a large portion of the United States, has been already responded to by many members of the profession. We should be glad to receive some histories of the yellow fever, especially as connected with the statistics of the disease in several cities of the South.

The extended circulation of the Examiner, particularly in the Southern States, renders these statements of great value to the profession; and the physician who publishes the results of his practice, (and all accurate accounts are interesting,) writes for his brethren scattered over a wide surface of country, and has the advantage of direct correspondence, with the permanence of a journal. It is often annoying to us, that we should be obliged to glean matter relative to the diseases most common in our own country from foreigners, who have seen them, as it were, only in passing, and have not become thoroughly or practically acquainted with their nature.

We have already published some interesting accounts of intermittent and remittent fevers; and we should be equally pleased to hear of the treatment and results of the same diseases in other localities, as well as of the yellow fever and dysentery.

There are still many points of interest, which can only be settled by careful collection of documents from numerous and scattered localities. For these diseases vary so much in their type, that they must be studied on a very extended scale, to accumulate any thing like positive data.

---

### INCREASE OF MEDICAL SCHOOLS.

The increase of medical schools induces some to fear that the profession is rapidly becoming overstocked with labourers who find no employment, and will never cease to regret their entrance upon a mode of life where every avenue seems blocked up. If the profession is to be regarded in a merely pecuniary light,—if, for the capital and labour expended, the physician is to look for the income which he might have gained by entering upon another career, in which the employment of capital is more direct, and its returns more abundant,—there is no doubt that physicians are too numerous. If a medical man is to look for no other reward than mere pecuniary profit, he will surely be disappointed. But there are other considerations which ought to be taken into the account. The social position which an educated physician claims, and the numerous sources of gratification which he derives from the exercise of a profession in which so many occasions arise of gratifying his kindly and benevolent feelings, are really part of his reward, and we well know that they are so regarded by most members of the profession.

Besides, in a country like ours, the passage from one occupation to another is so easy, that he who feels that he is illy fitted for the calling of a physician, or from circumstances is unable to pursue it in a way satisfactory to himself, can readily change it for another which offers greater profits, or is more congenial to his taste. The alarm which is sometimes expressed upon this subject is, we are sure, quite unnecessary. The maxim that no one is satisfied with his own vocation is at least as old as the days of Horace, and its justness will not be contested now.

The truth is, that a number of medical schools are necessary to supply the wants of the country, more especially as many of those educated for medicine either do not practice, or expect to follow their profession for a comparatively short period, leaving it afterwards for agriculture, or other similar pursuits.

The multiplication of schools is, however, attended with some temporary evils. The natural tendency of them may be to foist into the profession a certain number of incompetent persons, who, it is said, are urged to enter an institution to increase the number of its pupils. At least, this is an objection sometimes urged, and one not altogether without weight. But, if an institution is ever culpable of a grave error of this kind, its true and lasting interest is certainly not promoted by it, and it must soon cease.

The concentration of medical instruction in some two or three institutions is not practicable, nor indeed to be desired. The character of all our schools depends upon individual reputation, and in most respects they must be private institutions. This gives a freedom to their action which would be unattainable if very close restrictions were enforced; and an institution will rise or fall, first, according to its own merits, and secondly, according to the demand for instruction. If an institution is unnecessary, it will cease to be profitable, and may even be a source of expense to its members; this result will take place even if the faculty be thoroughly competent. This natural adjusting of the number of medical schools to the wants of the population, is, we are sure, the only natural and legitimate mode of getting over the difficulty. In fact, it is the mode adopted in every country to a greater or less degree.

It is stated sometimes that the number of schools in the United States is totally disproportioned to the number found in other countries: this is altogether an error. If we set down the number of schools at thirty, we shall probably rather exceed than fall short of the actual number. In England the number of schools is far greater; and although the population is more numerous than that of the United States, there is employment for a less number of medical men, for the plain reason that our extent of country is much greater, and many parts of it are less salubrious. Even on the continent of Europe the number of schools is much greater than would appear from a superficial examination. For example, in France, there are but three degree-conferring medical schools; but the number of secondary schools, where a portion, at least, of the period of study of each pupil is spent, is probably not much less than in the United States.

In England, the difficulty is removed by a very simple process. Degrees or licenses are granted by the University of London, the Colleges of Physicians, and Surgeons, and by the Apothecaries' Company: these institutions are not strictly bodies of teachers, although the teachers of many different schools belong to them. Should the number of medical schools tend to increase in this country, the natural check will doubtless be found in some similar arrangement; and we have no fears as to the result. In at least two Northern colleges, a beginning has already been made, and examiners or judges are added to the faculty at their examinations. This, or some other means, will doubtless arise from the necessity of the case, should the value of a diploma, from incautious conduct on the part of our medical schools, greatly sink below its accustomed estimate,—and we have no fear of overstocking the profession. A test will always be required, if not for permission to practise, at least for obtaining the confidence of the community. This test is, in the main, reputation as a skilful practitioner; but, besides this, examinations, and attendance on regular courses of lectures, will always have their value.

It is true, medical schools are sometimes founded where they are not really needed. This arises often from wrong and exaggerated estimates of the emoluments of teachers. With the exception of a very few institutions, medical schools do not pay nearly so well as the same employment of time and labour in the practice of the profession. Hence, as a general rule, a teacher is unwilling to trust to the fees of pupils for his sole support, and well he may be, for in most instances they afford but a slender livelihood. There are, therefore, but few who desire to separate the teacher from the practitioner; most men who are engaged in teaching, direct their attention at the same time to the practice of some department of the profession. There is then no real, still more, no necessary distinction between teachers and practitioners: the qualifications for success in teaching and practising a profession are, however, not precisely the same; and one may meet with but indifferent success in one pursuit, while he may have earned a well-deserved reputation in the other. That equal reputation is not more frequently attained in both these departments, arises from the many qualities which must be united in one individual. It is rare that the quickness of observation, coolness and rapidity of judgment, and decision of action, which are requisite to form a skilful practitioner, are united with the eloquence and methodical arrangement of thought necessary for an eminent teacher.

### DEATH OF DR. FRISBY, OF NATCHEZ.

We announce with great regret the death of one of our subscribers, from whose labours we anticipated many valuable contributions,—Dr. Asa Frisby, of Natchez. He fell a victim to the yellow fever, which has been so destructive during the present year in many of the Southern cities.

Dr. Frisby was a physician of uncommon promise, wedded to his profession, zealous in cultivating it, and fearless amid the dangers to which he seemed to be constantly exposed. During a residence of eighteen months at the Philadelphia Hospital, he contracted the typhus fever, which was at that time epidemic, and narrowly escaped with his life.

The duties at the hospital were most arduous, and were performed by him with a spirit above all praise; he fell at last a victim to another disease at least as formidable as epidemic typhus, and more insatiable in the number of its victims.

